# Does User Fee Removal Policy Provide Financial Protection from Catastrophic Health Care Payments? Evidence from Zambia

**DOI:** 10.1371/journal.pone.0146508

**Published:** 2016-01-21

**Authors:** Felix Masiye, Oliver Kaonga, Joses M Kirigia

**Affiliations:** 1 Department of Economics, School of Humanities and Social Sciences, University of Zambia, Lusaka, Zambia; 2 World Health Organization, Regional Office for Africa (WHO/AFRO), Brazzaville, Republic of Congo; University of Oxford, KENYA

## Abstract

**Background:**

Out-of-pocket payments in health care have been shown to impose significant burden on households in Sub-Saharan Africa, leading to constrained access to health care and impoverishment. In an effort to reduce the financial burden imposed on households by user fees, some countries in Sub-Saharan Africa have abolished user fees in the health sector. Zambia is one of few countries in Sub-Saharan Africa to abolish user fees in primary health care facilities with a view to alleviating financial burden of out-of-pocket payments among the poor. The main aim of this paper was to examine the extent and patterns of financial protection from fees following the decision to abolish user fees in public primary health facilities.

**Methods:**

Our analysis is based on a nationally representative health expenditure and utilization survey conducted in 2014. We calculated the incidence and intensity of catastrophic health expenditure based on households’ out-of-pocket payments during a visit as a percentage of total household consumption expenditure. We further show the intensity of the problem of catastrophic health expenditure (CHE) experienced by households.

**Results:**

Our analysis show that following the removal of user fees, a majority of patients who visited public health facilities benefitted from free care at the point of use. Further, seeking care at public primary health facilities is associated with a reduced likelihood of incurring CHE after controlling for economic wellbeing and other covariates. However, 10% of households are shown to suffer financial catastrophe as a result of out-of-pocket payments. Further, there is considerable inequality in the incidence of CHE whereby the poorest expenditure quintile experienced a much higher incidence.

**Conclusion:**

Despite the removal of user fees at primary health care level, CHE is high among the poorest sections of the population. This study also shows that cost of transportation is mainly responsible for limiting the protective effectiveness of user fee removal on CHE among particularly poorest households.

## Introduction and Background

Minimizing the risk of impoverishing health care payments is a valued policy goal in most health systems. In the context of many health systems in Sub-Saharan Africa, health systems depend heavily on user charges to supplement erratic and inadequate public funding to finance health service provision. Out-of-pocket payments (OOPPs) account for between 20% and 80% of total health spending in Africa [1]. As such, at household level, ability to meet user charges is a key determinant of access to health care as most health systems in Africa depend heavily on out-of-pocket payments by users. Therefore, it is hardly surprising that access to health care is very low among those residing in rural areas, uneducated, lowest wealth quintile and otherwise marginalized sections of society in many countries in Africa [2]. There is strong evidence that access to life-saving health care in many settings in Africa is constrained largely by financial burden imposed by out-of-pocket health care payments [3]. Furthermore, large sections of the populations in many parts of Africa are impoverished by health care payments or forgo treatment due to inability to mobilise resources needed to defray health care bills [4]. Consequently, inequities in financing remain a major policy challenge in Africa [5].

The financial burden imposed by out-of-pocket payments including user charges, on poor households has been a subject of health financing debate over the recent past [6,7]. The WHO has proposed that health expenditure be viewed as catastrophic whenever it is greater than or equal to 40% of a household's non-subsistence income, i.e. income available after basic needs have been met [8]. In an effort to reduce the financial burden imposed on households by user fees, some countries in Sub-Saharan Africa have abolished user fees in the health sector [9–11]. In other settings, financial protection policies are in form of selective or targeted exemptions or vouchers for the poor or other identified population groups.

The impoverishing effects of health care payments have been demonstrated to be a significant policy issue in many parts of Africa [12–14,4]. Studies have analysed the effect of out-of-pocket health payments on financial protection in the context of free public health care policy or local health insurance in Sub-Saharan Africa [15,16]. Most of these papers on the impact of user fees have focused on evaluating effects on access and utilization [17]. The limited available evidence shows a rather mixed association between user fee removal and financial protection against high cost of access to health care in developing countries. For example, in Uganda, examination of out-of-pocket payments before and after removal of user fee showed that the burden of out-of-pocket payments increased, especially among the poorest, following the removal of user fees [18]. The authors attributed this paradoxical finding to poor quality of free public health services which forced people to seek costly treatment in private facilities and in charging public hospitals. Similarly, findings in Burkina Faso show that poor quality of available TB treatment resulted in patients incurring cost in the private sector [19]. A study in South Africa showed that long distance to facilities represented a financial barrier and added a significant portion to the cost of seeking care [20]. These studies suggest that financial protection is closely related to the quality of the health-care system or the integrity of implementation of free health care policy [8]. Clearly, user fee removal policy is not a panacea to guarantee total financial protection.

Several countries in Sub-Saharan Africa have introduced user fee removal with a view to reducing financial barriers to access particularly among the poorest sections of the population. Zambia is only the fifth country, after South Africa, Uganda, Madagascar and Liberia, to implement abolition of user fees on all primary health services in the public sector. In other countries, user fee removal was targeted at either specific population groups [21,22] or for specific interventions such as maternal services [23]. The Zambian government abolished user fees on primary health care, beginning with rural areas in 2006 in rural areas and then extended the policy to urban areas in 2011. The policy states that all services provided in health facilities designated as primary healthcare facilities are free of charge. All health facilities classified under primary health care which include health posts, health centres and district-level hospitals in both rural and urban areas are officially classified as primary health care facilities. As such, all services provided under these facilities should be free. It is understood that such services include drugs, consultation, laboratory and other medical investigations, referral services, etc.

In this paper we show that 77% of patients who visited primary health care providers did not incur any out-pocket health expenses, confirming that the policy of user fee removal has been implemented nationwide. However, there is conceivably an interest to investigate the extent of financial burden among the one in five patients who reported making payments. The incidence of catastrophic out-of-pocket payments provides a key metric for assessing the valued health system goal of financial risk protection. This paper attempts to answer the question: Has the policy of free primary health care in Zambia provided protection from the risk of catastrophic health care payments to all who deserve it.

The main aim of the paper was to examine the extent and patterns of financial protection from fees following the decision to abolish user fees in public primary health facilities. The study focuses only on OOPPs on outpatient services conditional on having visited a health provider following an illness. OOPPs associated with a visit to a health provider include medical payments at a facility for consultation, drugs, medical investigations, as well as payments for transportation, food, and other costs incidental to a health visit. Our analysis uses a nationally-representative health expenditure and utilization survey conducted in 2014 by the Central Statistical Office (CSO), Ministry of Health (MOH) and the University of Zambia. Understanding the extent and patterns of catastrophic health payments in the context of free primary health care would provide lessons for countries that might be contemplating removal of user fees as an instrument for attaining universal health coverage (UHC) in Sub-Saharan Africa [24].

## A Description of the Zambian Health System

In this section we give some brief context of Zambian health system. Health care in Zambia is financed from four main sources namely, public revenue (25%), out-of-pocket payments (27%), donor funding (42%) and employer-based medical schemes (5%) [25]. It is clear that the Zambian health care system depends significantly on donors. The government abolished user fees in primary health care facilities. This entails that a person who visits a public primary health care facility does not face out-of-pocket expenditure at the point of use for services provided in that facility.

Health services are provided by four main players namely the government, faith-based not-for-profit providers, private-for-profit providers and traditional practitioners. According to recent surveys [26,27], about 90% of patients seek care in facilities owned and run by the government. Faith-based not-for-profit providers health facilities account for 6% of services provided while private-for-profit facilities provide care to approximately 3% of the population. Less than 2% of Zambians seek care in traditional providers. Although the data shows clearly that the public sector is the biggest health provider, there is nearly 30% of the sick who choose self-medication, most of whom bought over-the-counter drugs from drugs stores or pharmacies.

The public Zambian health delivery is structured as a three-tier pyramidal referral system-primary health care (health posts, health centers and district hospitals), secondary health care (provincial referral hospitals), and tertiary health care (teaching hospitals). At the bottom of the system are health posts. Health posts provide the first point of contact with the health system and provide largely preventive (immunization, growth monitoring, bed net distribution, intermittent presumptive treatment, family planning, etc.). In addition, the Ministry of Health supplies health posts with a very limited kit of drugs (what is referred to as health post drug kit) to be able to deliver only curative services (oral rehydration salts, uncomplicated malaria, respiratory infections with basic antibiotics, etc.). Health posts are operated by a public health specialist (called Environmental Health Technologists), a nurse or midwife. Due to a critical shortage of health staff, it is quite common to find a community health worker, a volunteer or even a watchman dispensing health services. Health posts do not have laboratories but do carry Rapid Diagnostic Tests and are able to dispense antimalarials. Unless a midwife is available, the health posts often refer antenatal care and deliveries to higher level health facilities.

The next level of care is the health centre which offer slightly more advanced interventions than health posts. In many cases health centres would have a laboratory. The cadres of health workers in a typical health centre would include clinical officers, nursing staff, midwives, environmental health technologist and laboratory technologists. The shortage of health staff especially in rural areas also limits the capabilities of health centres. Health centres also provide preventive services mentioned above. They also hold the cold chain on behalf of health posts in their neighbourhood because most health posts do not have energy sources. In terms of interventions, although most health centres do not have medical doctors, they have staff trained to provide antiretroviral therapy and treatment for non-drug resistant tuberculosis and uncomplicated malaria treatment. Most health centres offer only outpatient services.

Health centres refer complicated cases to a district hospital. District hospitals are still at the primary health care level. Here you find a general medical doctor, a pharmacist and greater laboratory capabilities and x-ray equipment. In addition to providing more complicated interventions, district hospitals offer basic surgery including caesarean sections. The next level of care is secondary level hospitals or general hospitals, i.e. the highest referral hospital in a province. These hospitals have consultant doctors and more advanced diagnostic services such as computed tomography (CT) scan. They also have an Intensive Care Unit. The highest level of care is the tertiary level hospital. Apart from offering the most specialized services available in the country tertiary hospitals also provide teaching services. In reality, the referral system does not function as expected. Most hospitals offer more primary health care than was intended. Partly, this is due to poor services at lower levels and a mal-distribution of lower level facilities.

## Methods

### Sample and data

The data used in this analysis was collected in the Zambia Household Health Expenditure and Utilisation Survey (ZHHEUS) in 2014, which was conducted by the Central Statistical Office, with support from the Ministry of Health and the University of Zambia. The national sample frame was used to select clusters from all ten provinces in Zambia and achieve national representation. Household clusters were selected using a two-stage sampling design after stratifying by rural-urban categories. The total of about 12,000 households, including some 60,000 individuals, in all ten provinces of Zambia was sampled. A 99.4% response rate was achieved. The survey was administered using face-to-face interviews by a team of research assistants, between January and March 2014.

Each member of a household was asked directly or through a household survey respondent about their illness experience and associated health care decision (e.g. if they visited a health provider), type of service sought and expenditure. Broadly, the survey instrument captured: (i) information about the household and its members; (ii) any experience of an illness or injury by any member of the household, (iii) health care utilization and resulting expenditures, and (iv) perceptions about quality of care received if applicable. It is important to note that the survey provided provision for respondents to report up to four health events and associated decisions and payments during the four weeks (for same illness or different illness). Furthermore, the survey asked about health expenditure including charges for consultation, drugs, medical investigations, and other fees incurred at facilities, as well as transportation costs and other costs related to a visit to a health provider.

#### Ethical considerations

Since the study did not involve collection of human samples, ethical exemption for this study was granted by the CSO under the provisions of the Census and Statistics Act Number 127 of the laws of Zambia. No identifying information of individuals or health institutions were collected in the survey. In observance of the ethical requirements, only participants aged at least 15 years were interviewed after giving written (signature or thumb print) informed consent.

#### Data on out-of-pocket health expenditure

In some studies on out-of-pocket health expenditure, only medical expenses such as drugs, consultation, investigations, medical procedures, and medical equipment (e.g. dentures, eye glasses, etc.) are included. However, some authors have argued that health expenditure should also capture costs such as transportation and any costs related to a visit to health provider [10,28]. In the survey on which this study is based, health expenditure data was defined to include items such as registration fee, consultation fee, drugs, medical investigations, transportation, food, and other items incidental to a visit to a health provider. This data allows us to isolate the role of travel costs in the analysis of financing burden of out-of-pocket health expenditure. Individuals were asked about any illness experience in the four weeks preceding the survey. Individuals were subsequently asked the following questions: choice of health care options (formal health provider, self-medication and did nothing), utilization of services, and out-of-pocket payments associated with a visit to a provider. Health expenditure which was captured for each member of a household was collapsed to a household level by summing health expenditure for all members of the household who may have reported expenditure during a health visit. In analysis of financial protection the household is often the unit of analysis. This study only considers visits to providers, mainly because user fee removal policy did not cover inpatient care.

### Data analysis

#### Incidence and intensity of catastrophic Out of Pocket Payments

Out-of-pocket health expenditure was related to a visit to a formal health provider following an illness by members of a household. As mentioned earlier, in this analysis we did not include expenditure related to hospitalisation, medical insurance payments or any other types of health expenditure. OOPP health expenditure was then aggregated to the household level by adding up OOPP health expenditure incurred by members of a household. All expenditure data, i.e. total household expenditure, subsistence expenditure and out-of-pocket health expenditure, which were reported for a period of one month, were converted to annual figures.

In line with literature, to measure the financial burden of OOPPs, we employ one of the most widely used measures of financial protection in health, namely catastrophic health expenditure. We adopted the WHO definition which defines catastrophic expenditure as a household experiencing out-of-pocket health expenditure exceed 40% of a household’s ‘capacity to pay’. ‘Capacity to pay is measured by total household expenditure minus expenditure on subsistence, essentially food [29]. Further, for purposes of comparison, we also calculated catastrophic health expenditure using the 10% threshold of total household expenditure.

For each household reporting an illness and visit to a health provider, we calculate ratio of health expenditure to total consumption expenditure. To calculate the incidence of catastrophic health payments we estimate the proportion of households that experienced health payments above the threshold. That is, we count the number of households whose ratio of out-of-pocket health expenditure to total household consumption expenditure exceeds 0.4, and then divide the headcount number by the total number of households who reported having a visit to provider. We provide these computations for each economic quintile as well as province. In addition to estimating the incidence of CHE, we show the intensity of the problem of CHE by calculating the CHE overshoot measure of CHE. The overshoot demonstrates the extent to which CHE exceeds the threshold [30].

#### Assessing effectiveness of free primary health care on reducing incidence of CHE

To assess the effectiveness of free primary health care policy in protecting patients against the risk of CHE, we test whether the likelihood of experiencing CHE is lower among patients who visited public primary health facilities. However, the dummy variable representing choice of public health facility might be endogenous. There is a reason to suspect the possibility that likelihood of CHE may be related with the type of provider chosen in a non-random fashion. For example, patients who visited public primary health facilities might be less prone to CHE compared to those who visited other types of providers such as public hospitals or private facilities. Endogeneity would result in biased and inefficient estimates [31]. We employed a two-stage residual inclusion (2SRI) model to test for endogeneity of choice of provider, particularly public primary health care [32]. In this model, the residuals would represent the unobserved factors that are correlated with both choice of provider and health care expenditure. In the first stage, we ran a logistic regression of choice of public primary health care on a set of covariates from which we saved residuals. In the second stage of the model we estimate the logistic regression of CHE on the same set of covariates plus the residuals from the first regression. The results show that the coefficient on the residuals is not statistically significant (coefficient = 53.351; p-value = 0.217).

### Limitations

This study has a few limitations. First, although respondents were asked about their illness and health care utilization and expenditure within the standard four-week window, there is a chance of recall bias. Surveys of this nature are always subject to some degree of recall bias as some respondents may not always remember accurately health events and the details of expenditure incurred. Second, another limitation of this study is the failure to link payment to quality of care received. As such, it is not possible to establish whether those who reported making OOPPs were seeking better quality of service. Third, the data did not permit us to explore the existence of unofficial/informal fees [33]. Despite the evidence that many individuals paid at facilities which are designated as primary health care, it is not possible to definitively point to informal payments. We believe that this is an important issue that remains open for future research. Fourth, relying on a cross sectional survey such as this one, we are unable to establish causality but rely on association. Finally, the survey did not ask respondents who resorted to self-medication about their health expenditure. Despite these limitations, the study has generated evidence that can be used to inform debate about increasing financial protection especially for the poorest.

## Results

### Prevalence of reported illness and health care choices

Table 1 shows the distribution of households reporting at least one illness by expenditure quintile and region of residence. People in the lowest quintile reported the highest proportion of illness episodes at 62% compared to 49% in the richest quintile. Incidence of illness episodes is higher in rural areas than urban areas.

**Table 1:** Proportion of Households reporting an illness by expenditure quintile and Region of residence.

	Number reporting an illness	Total number of household	% of households reporting an illness (95% CI)
**Quintile**			
Quintile1	1472	2372	62.1 (59.6 64.6)
Quintile2	1465	2368	61.9 (59.4 64.4)
Quintile3	1425	2369	60.2 (57.7 62.7)
Quintile4	1267	2369	53.5 (50.7 56.3)
Quintile5	1181	2369	49.9 (47.05 52.8)
**Region**			
Rural	4434	6880	64.4 (62.9 65.8)
Urban	2376	4967	47.8 (45.8 49.8)

Source: calculated from the ZAHHEUS dataset

Based on the data used in this analysis, it is estimated that a typical household in Zambia has an average number of 5 household members with a monthly average expenditure of about K1177 ($ 156) with 36% of it being food expenditure.

### Analysis of Out-of-pocket payments (OOPPs) for outpatient services

Table 2 presents summary statistics of out of pocket health expenditure incurred among those who sought formal outpatient care following an illness. The table shows the percentage of patients who did not pay anything for the care they received. Overall, about 77% of individuals who visited a health provider did not incur any out-of-pocket expenses. The proportion of patients who did not incur any health expenditure is higher in the rural areas and among the poorer households. The lowest quintile has the highest proportion of patients who did not incur any expenses. More people are more likely not to pay for health services in primary health facilities compared to private and third level facilities.

**Table 2:** OOP Health payments by region of residence, expenditure quintile and facility type.

	% reporting zero expenditure % (95% CI)	Mean expenditure (Kwacha) per visit for all who reported illness. Mean (95% CI)	Mean expenditure (Kwacha) per visit for those with positive health expenditure. Mean (95% CI)
**Region**			
Rural	83.2 (82.4 83.9)	21.2 (16.3 26.0)	126.0 (97.7 154.4)
Urban	72.2 (70.8 73.6)	40.9 (33.3 48.5)	147.8 (121.3 174.5)
**Quintile**			
Quintile1	88.0 (86.9 89.2)	11.0 (6.9 19.1)	84.07(62.1 106.0)
Quintile2	84.2 (86.9 89.2)	12.5 (9.3 17.6)	78.35(61.3 95.39)
Quintile3	77.7 (76.2 79.3)	25.7(17.4 33.9)	98.23(81.3 115.1)
Quintile4	74.6 (72.9 76.4)	27.6 (21.4 33.6)	112.56(93.6 131.5)
Quintile5	68.3 (66.2 70.4)	54.8 (43.4 66.1)	177.14(152.9 201.3)
**Facility type**			
Third level hospital	36.7 (31.3 42.2)	274.7 (188.5 360.8)	434.5 (303.2 565.9)
Public district hospital	60.6 (57.1 63.0)	62.4 (50.4 74.4)	156.4 (128.6 184.3)
Public rural health centre	71.1 (69.6 72.7)	22.6 (13.8 31.4)	78.4 (48.0 108.8)
Public urban health centre	55.0 (52.3 57.8)	39.8 (27.7 51.9)	88.5 (62.1 114.9)
Private facility	25.7 (19.7 31.7)	289.1 (153.5 424.9)	389.3 (208.9 569.8)
Public Health post	74.5 (72.1 78.0)	17.6 (12.2 22.9)	69.1 (48.9 89.2)
Other	96.3 (94.9 97.7)	2.3 (0.91 4.40)	19.8 (30.7 112.4)

Out-of-pocket health expenditure during a visit to a health provider is significantly higher in the richest quintile. On average an individual residing in urban area spent twice the amount spent by an individual in the rural area. The overall mean OOPP per visit was K14.90 (approximately US$2.50) for everyone who visited a provider, including those who reported spending nothing. If we consider only those who incurred positive amounts, the mean OOPP is significantly higher at K79.50 (US$ 13.03), implying that for those who had to incur OOPP, the financial burden of seeking care is quite considerable.

### Composition of OOP healthcare payments

**Fig 1:**
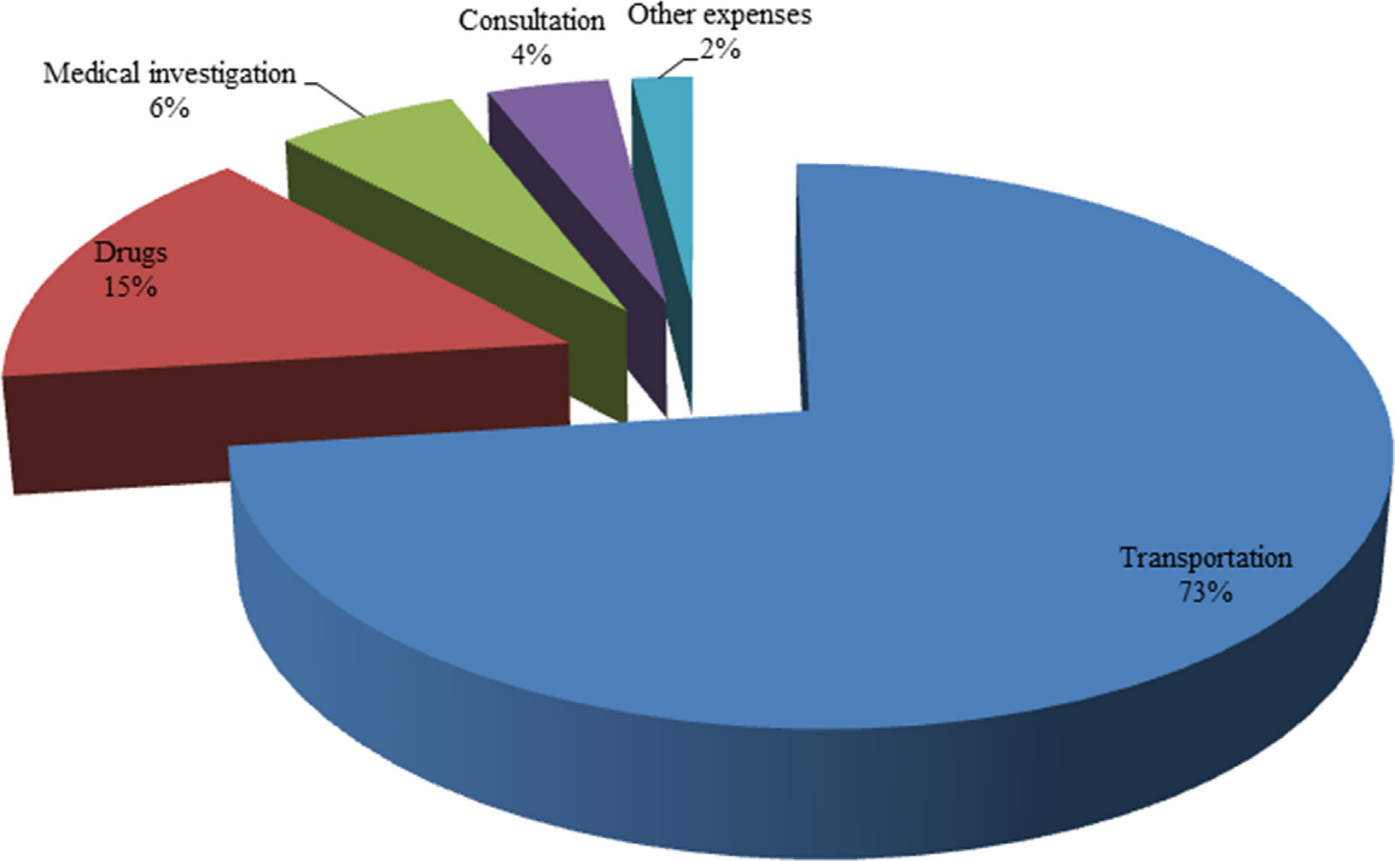
Composition of OOP healthcare payments.

To provide more information, we show the breakdown of OOPP in Fig 1. For those who incurred out of pocket health expenditure, 73% of it was spent on transport costs to a health provider. The rest of the expenses were treatment related costs with drugs accounting for 15% while medical investigation and consultation were reported at 6 and 4% respectively.

### Incidence of Catastrophic health expenditure for outpatient services

Table 3 presents results of the analysis of the incidence of catastrophic expenditure based on data from 6,810 households representing the 13,150 individuals who reported an illness four weeks prior to the survey. As the unit of analysis is the household, all health expenditure by individuals have been aggregated to the household level. The results shows the calculated incidence of catastrophic expenditure among households based on two mostly used threshold/cut-off levels; (i) equal to or greater than 40% of non-food expenditure and (ii) equal to or greater than 10% of total household expenditure. The national average incidence of catastrophic health expenditure ranges from 9.3% (using the threshold of 10% of total household expenditure) to 11.2% (using the threshold of 40% of non-food household expenditure). Using the definition of 40% of non-food expenditure, the distribution of CHE ranges from 16.3% among the lowest quintile to 4.8% among the richest quintile. The incidence of CHE is generally higher among the rural population. Finally, the incidence of CHE is highest among visitors to outpatient services in private facilities and higher level hospitals (20.8% and 32.0%, respectively).

**Table 3:** Incidence of Catastrophic Healthcare payments by region of residence, expenditure quintile and facility type (n = 6,810).

	Catastrophic healthcare expenditure for total OOP payments		Catastrophic healthcare expenditure for medical Payments	
	Threshold: 10% of total household expenditure n)	Threshold: 40% of non-food expenditure (n)	Threshold: 10% of total household expenditure (n)	Threshold: 40% of non-food expenditure (n)
**Region of residence**				
Rural	11.3% (503)	11.1% (494)	4.7% (212)	5.3% (234)
Urban	11.2% (267)	7.5% (179)	3.7% (88)	2.6% (62)
**Expenditure Quintile**				
Quintile1	14.8% (203)	16.3% (224)	10.4% (142)	12.6% (166)
Quintile2	8.9% (121)	9.2% (124)	2.7% (37)	3.6%(47)
Quintile3	12.1% (165)	10.9% (148)	3.8% (52)	3.1% (42)
Quintile4	10.4% (143)	8.1% (111)	2.4% (33)	1.5% (20)
Quintile5	10.1% (138)	4.8% (66)	2.6% (36)	1.4% (21)
**Facility type**				
2^nd^ and 3^rd^ level hospitals	42.4% (111)	32.1% (84)	11.8% (31)	8.0% (21)
Public district hospital	42.7% (157)	20.6% (159)	7.1% (55)	6.2% (48)
Public rural health centre	12.2% (208)	11.6% (205)	5.6% (119)	5.8% (122)
Public Urban health centre	12.9% (112)	8.6% (61)	4.2% (39)	2.6% (24)
Private facility	31.0% (58)	20.8% (39)	15.0% (28)	10.7% (20)
Public Health post	14.7% (94)	13.6% (102)	5.8% (49)	5.9% (50)
Other	6.4% (30)	4.9% (23)	1.9% (9)	1.9% (9)
**Overall**	11.2% (770)	9.3% (673)	4.2% (300)	4.0% (296)

### Catastrophic Healthcare payments for primary health care

Table 4 compares the incidence of catastrophic health expenditure among the population who visited primary health facilities with those who visited any type of health facilities (including primary health facilities). The incidence is higher among households who sought care at public primary health facilities (12.2% compared with 9.8%). This is the case without controlling for a particular household’s income or whether a particular household resided in rural or urban area. The incidence of CHE is higher both in terms of total OOPPs and medical categories of health expenditure (essentially, total OOPPs less transportation costs). This result appears somewhat paradoxical given that primary health care is supposed to be free. However, one possible explanation of this result could be that most of the people visiting primary health facilities are in the low income groups. The low household income means that even a considerable amount of health expenditure incurred can push the household into catastrophic expenditure. This results in a higher concentration of catastrophic payments among the poorest households.

**Table 4:** Incidence of Catastrophic Healthcare payments for primary health care.

	Catastrophic healthcare expenditure for total OOP payments		Catastrophic healthcare expenditure for medical Payments	
	Threshold: 40% of non-food expenditure (95% CI): all providers	Threshold: 40% of non-food expenditure (95% CI): public primary facilities only	Threshold: 40% of non-food expenditure (95% CI): all providers	Threshold: 40% of non-food expenditure (95% CI): public primary facilities only
**Region of residence**				
Rural	11.1% (10.1 12.4)	13.8% (11.6 16.0)	5.3% (3.2 7.4)	6.3% (4.0 8.6)
Urban	7.5% (5.9 9.1)	8.8% (5.6 12.0)	2.6% (-0.6 5.8)	2.6% (-0.6 5.8)
**Expenditure Quintile**				
Quintile1	16.3% (14.5 18.2)	20.8% (16.9 24.7)	12.6% (8.8 16.4)	14.7% (10.7 18.7)
Quintile2	9.2% (7.3 11.1)	11.9% (7.7 16.1)	3.6% (-0.3 7.5)	4.6% (0.2 9.0)
Quintile3	10.9% (8.9 12.8)	12.2% (8.2 16.2)	3.1% (-1.0 7.2)	3.0% (-1.0 7.0)
Quintile4	8.1% (6.2 9.8)	11.2% (6.9 15.5)	1.5% (-2.6 5.6)	1.6% (-2.6 5.8)
Quintile5	4.8% (2.8 6.8)	5.2% (1.1 9.3)	1.4% (-2.4 5.2)	1.6% (-2.5 5.7)
**Overall**	**9.8**% (8.9 10.7)	**12.2**% (10.4 14.0)	**4.3**% (2.6 6.0)	**5.1**% (3.2 7.0)

In Table 5 we present results of the logistic regression results which show the direction and strength of the association between the CHE and the set of independent variables at the individual level instead of group-level results shown in Table 3. CHE is significantly negatively associated with household expenditure with the likelihood of CHE nearly three times higher (OR = 2.9; p-value<0.00) in the poorest quintile compared with the richest quintile. The education attainment and employment status (either formally employed or not) of the head of household, and Sex of the patient were not found to be significantly associated with the likelihood of incurring CHE. Region of residence was found not to be significant. However, distance to the facility was associated with an increased likelihood of incurring CHE, which highlights the significance of distance in increasing cost of access to health care. Coming to the variable of interest, which is facility type, the results show that after controlling for income, distance and other covariates included in the regression, visiting a primary health care facility was associated with reduced likelihood of experiencing Catastrophic health care payments (OR = 0.68, p-value<0.00). In regards to the results in Table 4 which showed the incidence of CHE to be quite high among visitors to primary health facilities (due to a high concentration of the poorest at those facilities), Table 5 shows that after controlling for household expenditure and other factors, the likelihood of CHE is significantly lower among those who visited public primary health facilities.

**Table 5:** Logistic model estimation for likelihood of incurring CHE [incurred CHE = 1, 0 = otherwise].

Variable Name	Odds Ratio	Std. Err.
**Dependent variable**		
CHE(1 = Incurred CHE; 0 = Otherwise)		
**Independent variables**		
Sex(1 = Male)	1.046	0.071
Age	1.006***	0.002
Region (1 = Rural)	1.012	0.09
Distance	1.015***	0.002
Facility type (1 = Primary health facility; 0 = otherwise)	0.676***	0.062
quintile1	2.873***	0.47
quintile2	2.111***	0.345
quintile3	2.889***	0.446
quintile4	1.742***	0.273
Quintile5(reference category)	-	-
Employ(1 = Paid employment)	0.844	0.096
No formal education(reference category)	-	-
Primary	1.186	0.139
Secondary	1.217	0.155
Tertiary	1.118	0.219
Malaria(reference category)	-	-
Respiratory	1.441	0.233
Diarrhoea	1.069**	0.176
Headache	0.873	0.113
Fever	0.939	0.213
Other illness types	1.558***	0.123
_cons	0.055***	0.011

Number of obs = 7924; LR chi2 (19) = 247.89; Prob > chi2 = 0.000; Log likelihood = -2962.1; Pseudo R2 = 0.040.

*** p<0.01

** p<0.05

* p<0.1

### Severity of CHE

To give an indication of how much OOPPs exceeds the threshold, we calculated the catastrophic payment overshoot, as presented in Table 6. The overall catastrophic mean overshoot as well as the catastrophic positive mean overshoot are standard measures of the intensity of CHE. The overall overshoot measures the intensity of CHE in the whole sample (i.e. including households whose health expenditure may be below the CHE threshold) while the positive overshoot reflects the severity of CHE among those households classified as having CHE. Based on all the households with at least one sick person, we found the mean overshoot to be quite low at 0.68%. The intensity of catastrophic expenditure among households with catastrophic expenditure shows that on average, households exceeded the 40% threshold by 8%.

**Table 6:** Catastrophic healthcare expenditure Overshoot.

	Number of observations	Mean	95% CI
Mean overshoot	6810	0.68	[0.22 1.14]
Mean positive overshoot	69	8.00	[2.60 13.41]

## Discussion

In this paper we have assessed the level of financial protection from out-of-pocket payments on curative outpatient services in the context of free primary health care. Our analysis reveals mixed findings. One the one hand, our findings show that following the removal of user fees, a majority (83%) of patients who visited public health facilities benefitted from free care at the point of use. Adjusting for household economic wellbeing and other factors, the likelihood of facing CHE are significantly lower at primary health care level in the public sector. This result also confirms that implementation of user fee removal has been institutionalised in all public primary health facilities nationwide.

On the other hand, the study reveals significant inequality in the incidence and severity of CHE, even among patients who visited primary health care facilities. Significant inequality in the incidence of catastrophic health care payments is demonstrated when we compare across expenditure quintiles where 20.8% of households in the bottom expenditure quintile had to spend more than 40% of their non-food budget during a visit to a public primary health provider compared to 5.2% among the richest quintile. Further, the incidence of CHE is generally slightly higher among rural households, which reinforces the high burden of OOPPs that befalls on the rural poor [9]. A combination of higher costs of travel, lower cash incomes and poorer quality of services in rural facilities, among other factors, could explain this finding. In regards to the intensity of CHE, our estimate of mean CHE positive overshoot shows that households that faced CHE actually spent nearly half (48%) of their non-food budget on outpatient health care services. The foregoing analysis suggests that despite the removal of user fees at primary health care level, there is significantly less financial protection that is going to the poorest sections of the population.

A number of factors may explain the relatively high incidence of catastrophic expenditure even in the context where user fees on public primary health services have been removed. First, due to shortages of drugs or medical services, patients may be referred to buy drugs at retail drug stores or to go and have their medical tests done in private facilities. In this survey, it is shown that even patients who had visited public primary health care facilities reported spending out-of-pocket on drugs, medical tests and other medical items, suggesting that inadequate care in public primary health facilities could be forcing individuals to seek more or better care elsewhere. Studies have found this phenomenon in Zambia [34,35]. Second, some patients incur considerable expenses in travel costs. These costs would be invariably significantly greater for the poorest sections who tend to live farther from health facilities [20]. A third possibility is informal charges whereby health workers could have sold drugs or received bribes to perform medical tests. Unfortunately, the data in the survey did not allow us to explore this possibility. Finally, a patient may have visited a private health facility or a public facility at a level higher than primary health care (i.e. charging) as opposed to seeking free care at a public primary facility. For example, patients may bypass free public primary health facilities on account of quality considerations or convenience of location of a facility [36].

The incidence of CHE in Zambia is generally similar to what has been reported elsewhere in Sub-Saharan Africa [10,37].Using the 40% of non-food expenditure threshold, it is reported that 11% of Kenyan households experiences CHE during a visit to an outpatient health facility [10]. Other studies have found much higher incidence of CHE. For example, a study in Nigeria reported that on 27% incidence of CHE associated with outpatient visits [9]. Another study that provides some context for this study is a multi-country study which estimated that 2.9% of households in Zambia incurred CHE [2]. Direct comparison of our results with this study is limited by the differences in methodology, particularly with regard to how health expenditure was measured. Furthermore, our study focuses only on OOPPs for outpatient treatment as opposed to all health expenditure incurred during a period of time. Partly, the absence of user fees and the high preference for public health services explain why the incidence of CHE is lower than elsewhere.

## Conclusion

This study provides an assessment of the incidence and patterns of CHE following the removal of user fees on outpatient services in Zambia. The evidence shows that the policy of free primary health care services has benefited a majority of patients with free care at the point of use. At the individual level, after controlling for per capita household expenditure and other covariates, the likelihood of facing CHE is lower at primary health care facilities. However, this study has also found that overall, a critical 10% of the population experienced catastrophic health payments on outpatient visits. More importantly, considerable inequalities in the incidence of CHE are shown to exist in Zambia. Despite the policy of free primary health care services in the public sector, one in five patients in the poorest quintile faced CHE. Transportation costs are responsible for making many households vulnerable to catastrophic health expenditure especially among the rural poor. Clearly, financial risk protection of user fee removal seems to have benefitted those households with relative economic means and residing within reasonable distance of a public primary health care facility. Investment in more health infrastructure and better quality of care will be crucial for promoting greater financial protection against CHE as Zambia embarks on her quest for universal health coverage.

Findings from this study raise a number of implications for policy. For example, the fact that we observe OOPPs in public primary health care facilities raises a need to evaluate implementation of free primary health care policy. Of particular interest would be to establish if some of these payments are informal charges. Further, this study also shows that 10% of those who did not seek care cited unaffordable user fees which suggests that sensitisation of people that primary health care is free in the public sector is necessary. In addition, to alleviate the cost of care attributed to transportation costs, greater investment in physical access is needed to bring health care closer to people so that the benefits of free primary health care can reach more of the poor. Finally, investments in improving quality of care especially in rural facilities is likely to reduce the financial risk of CHE among the poor who are forced to spend on services unavailable in public facilities.
